# Highly Specific Detection of Myostatin Prodomain by an Immunoradiometric Sandwich Assay in Serum of Healthy Individuals and Patients

**DOI:** 10.1371/journal.pone.0080454

**Published:** 2013-11-15

**Authors:** Astrid Breitbart, Gesine M. Scharf, David Duncker, Christian Widera, Jens Gottlieb, Arndt Vogel, Sebastian Schmidt, Gudrun Brandes, Hans-Gert Heuft, Ralf Lichtinghagen, Tibor Kempf, Kai C. Wollert, Johann Bauersachs, Joerg Heineke

**Affiliations:** 1 Klinik für Kardiologie und Angiologie, Medizinische Hochschule Hannover, Hannover, Germany; 2 Klinik für Gastroenterologie, Hepatologie und Endokrinologie, Medizinische Hochschule Hannover, Hannover, Germany; 3 Klinik für Pneumologie, Medizinische Hochschule Hannover, Hannover, Germany; 4 Institut für Zellbiologie im Zentrum Anatomie, Medizinische Hochschule Hannover, Hannover, Germany; 5 Institut für Transfusionsmedizin, Medizinische Hochschule Hannover, Hannover, Germany; 6 Institut für Klinische Chemie, Medizinische Hochschule Hannover, Hannover, Germany; Scuola Superiore Sant'Anna, Italy

## Abstract

**Background:**

Myostatin is a muscle derived factor that functions as a negative regulator of skeletal muscle growth. Induction of myostatin expression was observed in rodent models of muscle wasting and in cachectic patients with cancer or pulmonary disease. Therefore, there is an increasing interest to use serum myostatin as a biomarker.

**Methods:**

We established an immunoradiometric sandwich assay (IRMA), which uses a commercially available chicken polyclonal, affinity purified antibody directed against human myostatin prodomain. We determined the serum concentrations of myostatin prodomain in 249 healthy individuals as well as 169 patients with heart failure, 53 patients with cancer and 44 patients with chronic pulmonary disease.

**Results:**

The IRMA had a detection limit of 0.7ng/ml, an intraassay imprecision of ≤14.1% and an interassay imprecision of ≤ 18.9%. The specificity of our assay was demonstrated by size exclusion chromatography, detection of myostatin by Western-blotting and a SMAD-dependent transcriptional-reporter assay in the signal-rich serum fractions, as well as lack of interference by unspecific substances like albumin, hemoglobin or lipids. Myostatin prodomain was stable at room temperature and resistant to freeze-thaw cycles. Apparently healthy individuals over the age of 55 had a median myostatin prodomain serum concentration of 3.9ng/ml (25^th^-75^th^ percentiles, 2-7ng/ml) and we could not detect increased levels in patients with stable chronic heart failure or cancer related weight loss. In contrast, we found strongly elevated concentrations of myostatin prodomain (median 26.9ng/ml, 25^th^-75^th^ percentiles, 7-100ng/ml) in the serum of underweight patients with chronic pulmonary disease.

**Conclusions:**

We established a highly specific IRMA for the quantification of myostatin prodomain concentration in human serum. Our assay could be useful to study myostatin as a biomarker for example in patients with chronic pulmonary disease, as we detected highly elevated myostatin prodomain serum levels in underweight individuals of this group.

## Introduction

Myostatin is a protein of the TGF-β family secreted mainly by skeletal muscle fibres but also by adipocytes and cardiac myocytes [1,2]. It is an evolutionary conserved, strong negative regulator of skeletal muscle growth. Myostatin is produced within the cell as the precursor molecule pre-promyostatin (a disulfide-linked homodimer) and is converted to promyostatin by removal of the N-terminal 24-amino acid signal peptide. Subsequently, a furin protein convertase cleaves the promyostatin at amino acids 240-243 to generate an N-terminal fragment (27.7kDa, called myostatin prodomain) and a biologically active C-terminal fragment (12.4kDa, called myostatin ligand) [1,2]. After cleavage, myostatin ligand and prodomain are secreted by the cell and stay non-covalently associated with each other in a “latent complex” that also constitutes the main form of myostatin found in serum [3,4]. In the “latent complex”, which consists of two molecules of each myostatin prodomain and ligand and therefore is about 80kDa in size, the prodomain inhibits the activity of the myostatin ligand [1,2]. Full activation of myostatin occurs when the prodomain is cleaved by members of the BMP1/tolloid family of metalloproteinases leading to release of the myostatin ligand from the complex. 

Myostatin ligand binds to the activin receptor IIB at its target cells, which is also bound by the TGF-β family members activin-A and GDF11 [1,2]. Myostatin not only plays a prominent role as negative regulator of skeletal muscle size during physiological pre- and postnatal growth, but also during disease in adult life, when its expression is induced in cardiac or skeletal muscle of rodents with heart disease or cancer as well as in patients with cardiomyopathy, chronic obstructive pulmonary disease and some selected forms of cancer [5-13]. Increased serum levels of the myostatin ligand were found to accompany enhanced tissue expression in mice with heart failure, but it is not known whether this is also the case in patients [6]. Because genetic, antibody or decoy receptor mediated inhibition of myostatin reverses cachexia in mice with different diseases and because cachexia in chronic disease is associated with a particularly poor prognosis in humans [6,14-18], therapeutic strategies to inhibit myostatin signalling in patients are being developed [19]. However, in order to be able to diagnose myostatin induced cachexia, to identify patients at risk for muscle wasting or bad prognosis and to guide therapy, it would be necessary to determine the serum myostatin concentration in a reliable, very specific and high-throughput manner. The development of such an assay has been proven to be difficult and only recently one competitive immunoassay, which was designed to measure the precursor molecule promyostatin, has been commercially released and is currently mainly used for this purpose [20]. However, for example in heart failure patients, very variable results have been reported with this assay: While two studies found a significant increase of promyostatin in serum, two other groups reported the opposite in similar cohorts of patients as compared to healthy individuals [20-23]. These inconsistent results might suggest technical shortcomings of the assay. In addition, it is currently not believed that the precursor molecule promyostatin can be found in serum. In order to get a more meaningful picture of the systemic myostatin status it might be reasonable to separately determine the serum concentration of both the myostatin ligand and also the myostatin prodomain, which acts as strong inhibitor of the ligand. While a specific assay to detect the myostatin ligand has recently been introduced [24], an assay with proven specificity and adequate assay characteristics has not been available for the detection of the myostatin prodomain.

Here, we established a highly specific and reliable immunoradiometric sandwich assay for the quantification of myostatin prodomain in human serum. We assessed the concentration of myostatin prodomain in the serum of 249 healthy individuals, 169 heart failure patients (the biggest cohort reported for myostatin so far) as well as 53 cancer patients and 44 patients with chronic pulmonary disease. In contrast to some previous reports, we found no increase of serum myostatin prodomain in patients with heart failure, but for the first time demonstrated a profound rise of myostatin prodomain in serum of underweight patients with pulmonary disease.

## Materials and Methods

### Ethics statement

This study was approved by our institutional review board (“MHH Ethikkommission”). All individuals and patients were recruited at the Hannover Medical School and all gave written informed consent.

### Materials

The polyclonal chicken anti-human myostatin prodomain antibody (RD183057050) used in our IRMA was obtained from Biovendor. Serum promyostatin was measured with the Myostatin ELISA kit (K1012, Immundiagnostik). 

### Myostatin prodomain sandwich IRMA

Tubes (Nunc Maxisorp) were coated with 0.5µg polyclonal myostatin prodomain antibody diluted in 0.2ml sodium carbonate buffer (0.1M, pH 9.5) overnight. After three washing steps with phosphate buffered sodium chloride (40mM sodium hydrogen phosphate, 150mM sodium chloride, 0.1% Tween 20, pH 7.4), tubes were blocked with casein buffer (10g/l casein, 5g/l BSA, in 0.1M sodium carbonate buffer, pH 9.5) for 1 hour at room temperature. After two washing steps, 100µL assay buffer (40mM sodium hydrogen phosphate, 150mM sodium chloride, 0.1% sodium azide, 5g/L bovine serum albumin, 10ml/L mice serum, pH 7.4) and 100µL serum sample were added to each tube and incubated overnight at 4°C. The polyclonal detection antibody (5µg) was labelled with ^125^I (5MBq) using the chloramine-T method and was extracted by size exclusion chromatography on a sephadex G-25 column [25]. The samples were removed from the tubes, which were washed three times before 200µL of the detection antibody (diluted in assay buffer at 100µg/L) were added. After overnight incubation at 4°C and three washing steps, the samples were measured in a gamma-counter (LKB Wallac 1261). Each sample was measured in duplicate and the myostatin concentration was calculated with a calibration curve (included in each run) containing several concentrations from 0 to 100ng/ml.

Assay linearity was determined by serial dilutions of 6 serum samples with above normal concentrations. The assay showed linear results for the serum dilutions with deviations of the measured and calculated concentrations <25% in each sample for concentrations ranging from 2 to 485ng/ml. 

The specificity of our IRMA was confirmed by size-exclusion chromatography, Western-blot and the determination of myostatin triggered SMAD dependent CAGA-luciferase activity as described previously [26-28] and in detail in the Materials & Methods S1.

### Serum samples

We collected serum from apparently healthy blood donors from the blood donor center, from patients with chronic stable heart failure (outpatient arrhythmia clinic), from patients with gastrointestinal or hepatic cancer with current weight loss (outpatient gastro-oncological clinic) as well as underweight patients with chronic pulmonary disease (from the outpatient pneumological clinic). More information can be found in Table 1, Materials & Methods S1 and Tables S1, S2, S3.

**Table 1:** Clinical and biochemical characteristics of apparently healthy individuals and patients included in this study.

	**Healthy ≤ 55 years**	**Healthy > 55 years**	**Heart Failure**	**GI-Cancer**	**Chronic Pulmonary Disease**
**Number of individuals**	63	186	169	53	44
**Age, years**	25 (22-40)	60 (57-64)	70 (62-75)*	64.2 (52-70.5)	36.2 (26-50)^#^
**Male Sex %**	57.1	66.7	81.7 *	67.9	31.8^#^
**BMI, kg/m^2^**	N/A	N/A	27.9 (25-32)	23.4 (22-27)	17.5 (16.9-18.3)^§ §^
**Weight loss, kg**	N/A	N/A	0 (2-(-1))	10.0 (6-14)^§ §^	0 (0-(-2))
**Creatinine, µmol/l**	N/A	N/A	94.5 (78-122)^§ §^	69.0 (54-79)^§ §^	45.5 (41-59)^§ §^
**CRP, mg/l**	N/A	N/A	2.2 (1-5)	15.0 (5-42)^§^	4.0 (1-44)
**NT-proBNP (ng/l)**	N/A	N/A	698 (283-1296)^§ §^	162 (83-425)^§ §^	78.1 (52-133)^§ §^
**MSTN-Prodom., ng/ml**	6.0 (1-22)	3.9 (2-7)	2.6 (1-6)**	1.1 (0.4-6.7)**	26.9 (7-100)^# #^

Data are presented as the percentage or the median (25^th^-75^th^ percentile). Weight loss is indicated for a period of 6 months in heart failure and 12 months in patients with hepatic or gastrointestinal cancer (GI-Cancer). * p<0.05 or ** p<0.001 vs. healthy individuals > 55 years of age; # p<0.05 or ##p<0.001 vs. healthy individuals ≤ 55 years of age; § p<0.05 or §§ p<0.01 vs. other groups in which a value was obtained. BMI = Body Mass Index. CRP = C-reactive protein. NT-proBNP = N-terminal prohormone of brain natriuretic peptide. MSTN-Prodom. = Myostatin Prodomain. N/A = not available.

### Statistical analysis

Data lower than the detection limit were set as mean between zero and detection limit. We tested for differences between the median of two distinct groups with the Mann-Whitney-U-Test. Correlations between different variables were tested with the Spearman Rank Correlation Test. 

## Results

### Myostatin prodomain IRMA

**Figure 1:**
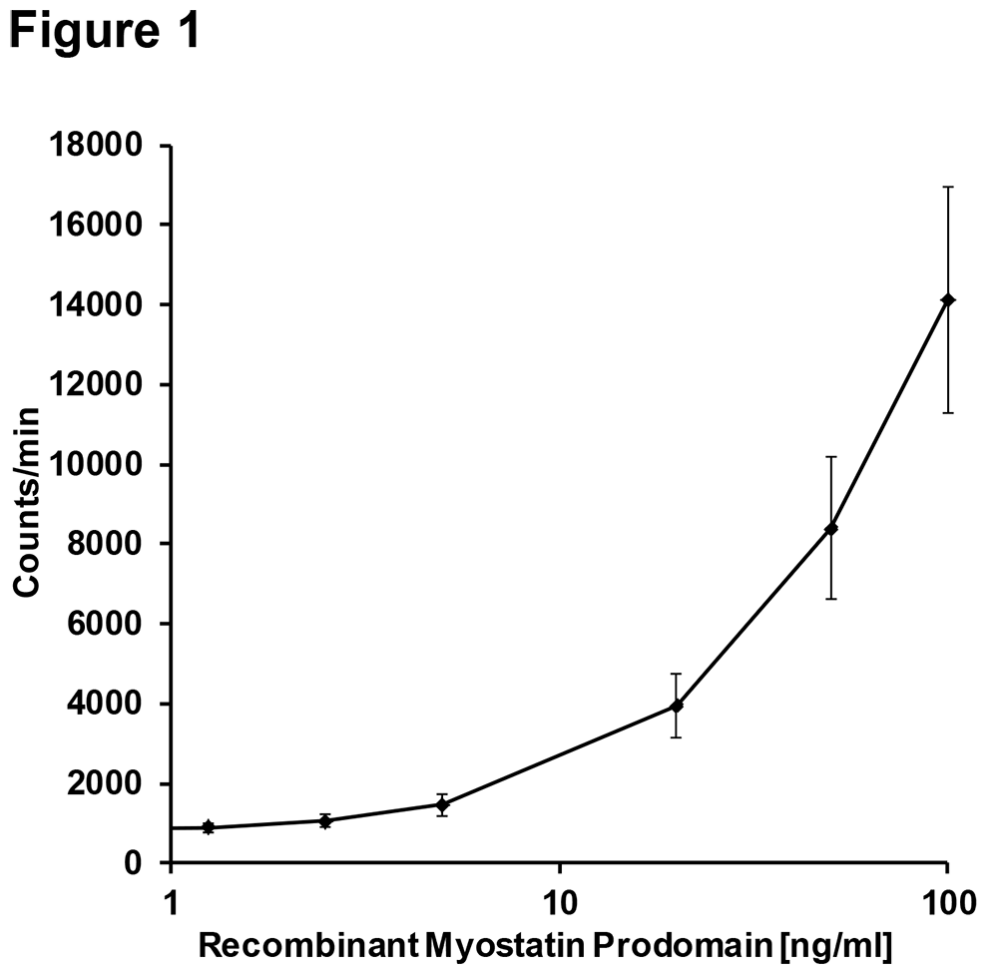
Mean calibration curve of the myostatin prodomain immunoradiometric sandwich assay. The mean calibration curve of 20 independent assay runs is shown. The x-axis is displayed as logarithmic scale. The values on the y-axis are presented as mean with the 95% confidence limits.

The calibration curve consisted of a zero sample and six non-zero samples covering a range from 1.25-100ng/ml. A mean calibration curve of 20 different assay runs is shown in Figure 1. The limit of detection of the assay was 0.7ng/mL and the limit of blank was 0.37ng/ml, both were determined according to international guidelines following the CLSI EP17-A protocol [29]. The functional sensitivity (=limit of quantification) was 1.25ng/ml, determined as the lowest concentration with an imprecision <20% in 20 repetitive measurements of human serum samples, with different lots of tracer and reagents over a time period of more than 8 weeks like suggested by Spencer and colleagues [30]. The within-run (intraassay) imprecision ranged from 3.7-14.1% and was determined in 10 parallel duplicate measurements of 11 serum samples containing 1.5-100.4ng/ml myostatin prodomain. The total (interassay) imprecision ranged from 10.0-18.9% and was determined in 10-13 assay-runs of 7 pooled serum samples containing 7.4-58ng/ml myostatin prodomain on different days, by two different operators and with different lots of antibodies, tubes and tracers. The repeatability standard deviation was determined for 3 pooled serum samples containing low (3.47ng/ml), medium (8.14ng/ml) and high (25.51ng/ml) myostatin prodomain levels. It ranged from 0.36ng/ml (low level sera) to 2.11ng/ml (high level sera), while the within laboratory precision standard deviation ranged from 0.96ng/ml for low level sera to 3.22ng/ml for high level sera, all values were determined according to the CLSI EP05-A2 protocol (appendix c) [31]. 

**Figure 2:**
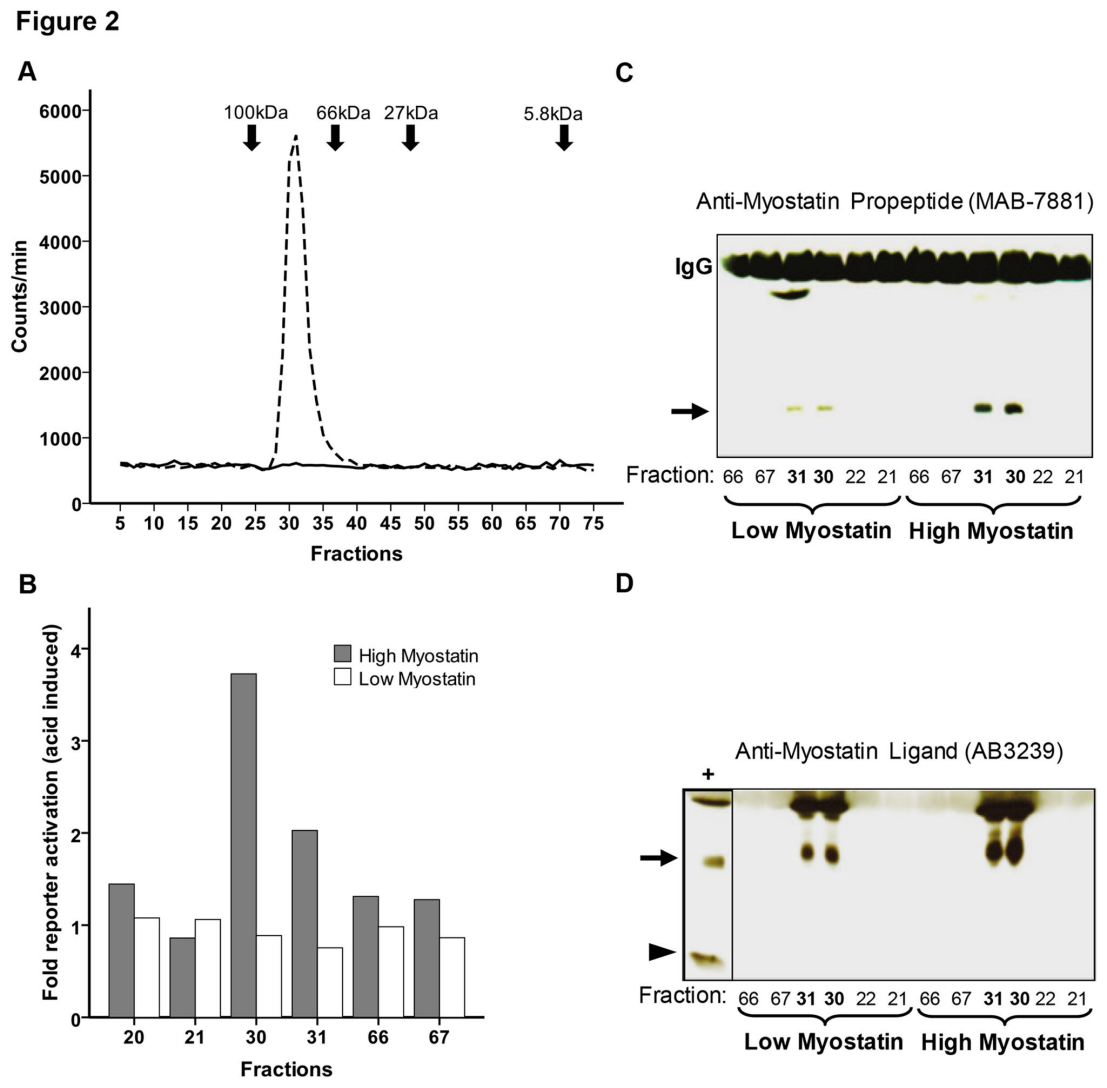
Specificity of the myostatin prodomain immunoradiometric sandwich assay. **A** Size-exclusion chromatography of two serum samples analyzed for myostatin prodomain. The dotted line represents the serum with high myostatin prodomain concentration (1629ng/ml), while the continuous line represents the serum with the low myostatin prodomain concentration (1.37ng/ml). B SMAD (CAGA) luciferase reporter assay showing acid induced myostatin activity in different serum fractions of one serum with high and one with low myostatin concentration as determined by our IRMA C Western-blot showing myostatin prodomain levels (arrow, 25kDa) as detected by the propeptide (=prodomain) specific antibody MAB-7881 (R&D systems). Low/high myostatin denotes serum with low and high myostatin prodomain concentrations. D Western-blot showing myostatin ligand dimers (arrow, 25kDa) as detected by the myostatin ligand specific antibody AB3239 (Millipore). + denotes positive control (recombinant myostatin ligand, R&D systems), the arrow head marks the localization of monomeric myostatin ligand (12.5 kDa). Low/high myostatin denotes serum with low and high myostatin prodomain concentrations.

In order to assess the specificity of our assay for the detection of myostatin prodomain in serum, we first fractionated serum by size exclusion chromatography and measured the prodomain concentration in 75 subfractions. As shown in Figure 2A, this procedure revealed a single peak of high myostatin prodomain concentrations in fractions 26-35 from a human serum sample with high prodomain abundance (1629ng/ml), but not in a serum sample with low abundance (1.37ng/ml). Two additional serum samples with high myostatin prodomain concentrations were analyzed accordingly and also revealed a single signal peak in fractions 26-35 (Figure S3). The size of the protein that produced the signal in the IRMA was between 66-100kDa, which is compatible with the size of the latent complex of myostatin prodomain and ligand (80kDa). In order to verify that the signal in our IRMA was indeed produced by the myostatin latent complex, we analyzed selected fractions of both serum samples (the one with high and the one with low concentrations from Figure 2A) with an assay that determines myostatin ligand triggered activation of SMAD protein dependent transcription (CAGA luciferase assay) as well as by Western-blot with two different antibodies, of which one detected the myostatin prodomain (also called propeptide), while the other detected the myostatin ligand. Figure 2B shows that acid treatment to release the myostatin ligand from the latent complex resulted in highest luciferase reporter activity in fractions 30 and 31, which also contained the highest myostatin prodomain concentration in the IRMA, and this activity was markedly higher in the serum with the higher myostatin abundance. Accordingly, as depicted in Figure 2C and D and detected by Western-blot, serum fractions 30 and 31 included both the myostatin prodomain as well as the ligand and both were more abundant in the fractions from the serum with the high myostatin prodomain concentration (as determined by the IRMA). Prodomain and ligand could not be detected in fractions outside the signal containing serum fractions 26-35 (Figure 2C, D and data not shown). When we analyzed the serum fractions with the commercial immunoassay, which was designed to detect the precursor promyostatin, no distinct peak of high myostatin concentration could be detected (Figure S1). In addition, the commercial assay could not detect recombinant myostatin ligand and only weakly detected the prodomain (Figure S2A, left). Detection of the prodomain or ligand was also not improved by their combination (to mimic latent complex formation, see Figure S2A, right). Furthermore, we determined serum myostatin concentrations in parallel with our prodomain IRMA as well as with the promyostatin immunoassay in the 169 heart failure patients included in our study and found no correlation between the results of both methods (data not shown).

In order to analyze for interference of prevalent substances with the sandwich IRMA, we added lipid particles (up to 5mmol/L), bilirubin (up to 300µmol/L), albumin (up to 40g/L), hemoglobin (up to 5g/L) and urea (up to 2g/L) to three different serum samples at normal and above normal concentrations. The deviation of myostatin prodomain concentration with and without added substances was <20% in all measurements. Similarly, the mature myostatin ligand also did not interfere with our IRMA (Figure S2B), which was designed to selectively detect the myostatin prodomain. 

For the evaluation of the preanalytic performance, two serum aliquots were stored at room temperature for 48h. The radioactive quantification showed no loss of signal compared to samples that were immediately frozen (105.9-111.7% to 100% at immediate freezing). Furthermore, 5 samples with low, normal and above normal concentrations of myostatin prodomain were frozen and thawed for four times, which also did not distinctly influence the calculated myostatin prodomain concentrations (87.4-118.4% to 100% at baseline).

### Myostatin prodomain in healthy individuals

**Figure 3:**
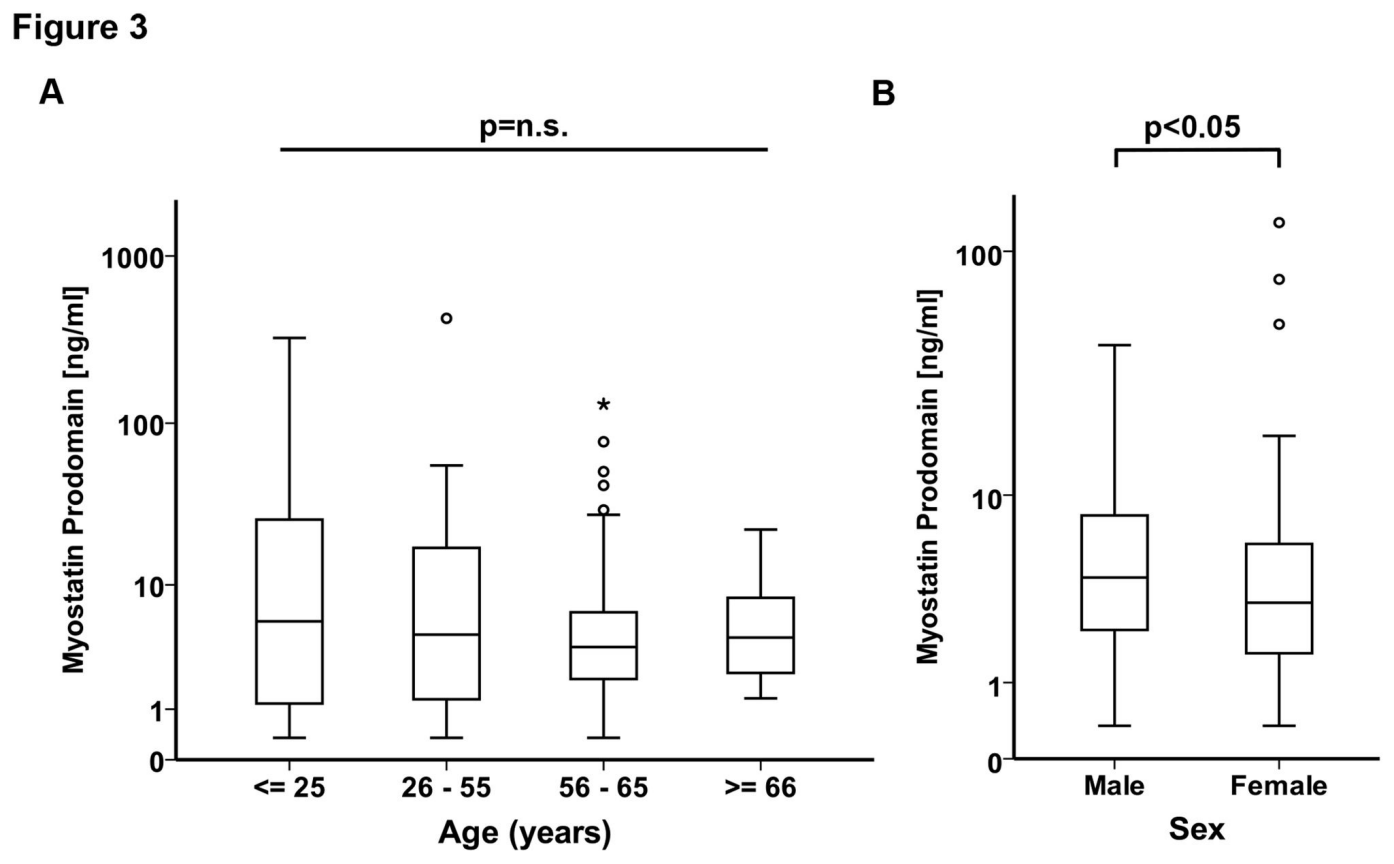
Myostatin prodomain serum levels in healthy individuals. **A** Myostatin prodomain serum concentrations in apparently healthy individuals ≤25 (n=33), 26-55 (n=46), 56-65 (n=138) and ≥66 (n=32) years of age. **B** Myostatin prodomain serum concentration in apparently healthy male and female individuals ≥55 years of age. Data are presented as box (25^th^ percentile, median, and 75^th^ percentile) and whisker (10^th^ and 90^th^ percentiles) plots.

We found that the myostatin prodomain serum concentrations among 249 apparently healthy blood donors did not significantly differ between age groups from under 25 years to over 66 years (Figure 3A). The median prodomain concentration in the blood donors under 56 years of age (n=63) was 6.0ng/ml (25th-75th percentiles 1-22ng/ml), and 3.9ng/ml (25th-75th percentiles 2-7ng/ml) in the apparently healthy blood donors older than 55 years (n=186, this difference was not statistically significant). The values were normally distributed in the 249 subjects upon logarithmic transformation. Interestingly, females over the age of 55 had a significantly lower prodomain serum concentration than males in the same age group (Figure 3B). 

### Myostatin prodomain in chronic heart failure patients

Because increased myostatin levels were detected in the myocardium and increased as well as decreased levels of myostatin have been reported in serum of mice and humans with heart failure, we wanted to analyze the concentration of myostatin prodomain in serum of patients with chronic heart failure (CHF) with our newly established IRMA. For this purpose, we recruited 169 patients with stable CHF, of which 117 (69.2%) were in New York Heart Association (NYHA) class II and 52 (30.8%) were in NYHA class III (Table 1 and Table S1). Echocardiography results obtained from the patients´ medical record indicated severely impaired ejection fractions (EF) in 109 patients (64.5%), moderately impaired EF in 29 patients (17.2%), slightly decreased EF in 7 patients (4.1%), while 6 patients (3.6%) had no detectable systolic dysfunction (from the rest of the patients no information on the EF was available).

**Figure 4:**
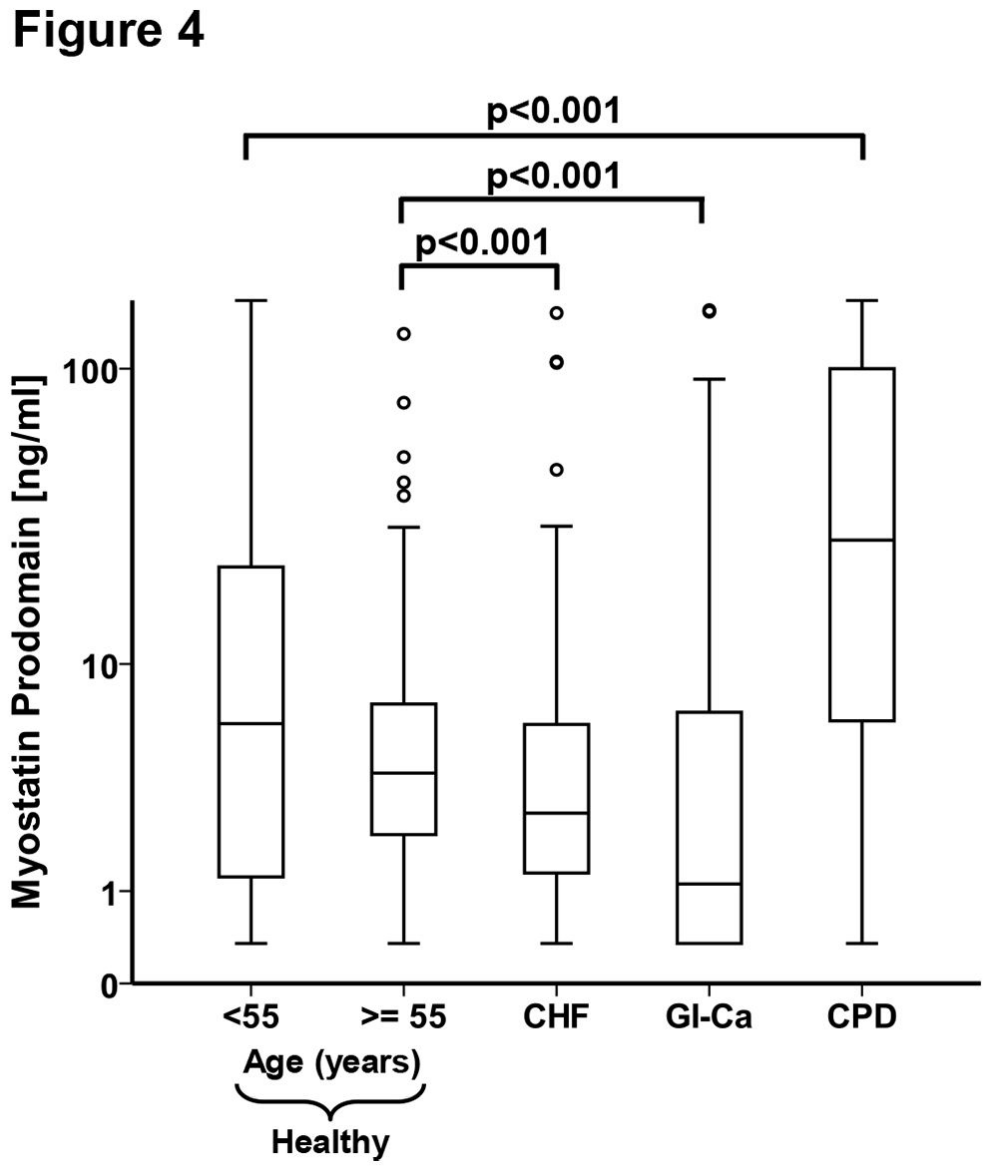
Myostatin prodomain serum levels in patients compared to healthy individuals. Myostatin prodomain serum concentrations of apparently healthy individuals <55 (n=63) or ≥55 years of age (n=186), in patients suffering from heart failure (CHF, n=169), gastrointestinal or hepatic cancer (GI-Ca, n=53) and chronic pulmonary disease (CPD, n=44). Data are presented as box (25^th^ percentile, median, and 75^th^ percentile) and whisker (10^th^ and 90^th^ percentiles) plots.

Myostatin prodomain was detectable in 143 (84.6%) of these 169 heart failure patients. Interestingly, the median myostatin prodomain concentration was 2.6ng/ml (25th-75th percentiles 1-6ng/ml) and was significantly lower compared to the older healthy group (p<0.001, see Figure 4). As expected, the serum concentrations of NT-proBNP (Table 1) and GDF15 (not shown), two biomarkers of heart failure, were elevated above the range generally observed in healthy individuals. We did not detect any significant correlation of myostatin prodomain with age, BMI, creatinine, GDF15, NT-proBNP, systolic blood pressure or NYHA class in this cohort (data not shown). The myostatin prodomain serum concentration was positively correlated to serum CRP (rho=0.156, p=0.043).

### Myostatin prodomain in patients with gastrointestinal cancer or chronic pulmonary disease

The lack of increased serum myostatin prodomain in the heart failure patients might reflect the absence of any pro-cachectic activity in these patients, which neither reported any current weight loss nor suffered from underweight (the median body-mass-index, BMI, was 27.9kg/m^2^). Therefore, we wanted to analyze the myostatin abundance in serum of patients suffering from acute or chronic weight loss. For this purpose, we first analyzed serum myostatin prodomain in 53 patients with gastrointestinal or hepatic cancer, who were undergoing acute weight loss (median weight loss within 12 months or less: 10.0kg, 25th-75th percentiles 6-14kg, see Table 1 and Table S2). The myostatin prodomain was detectable in 29 of 53 patients in this cohort (54.7%) and, interestingly, the median myostatin prodomain concentration was not elevated, but significantly lower compared to the older control group (median 1.1ng/ml; 25th-75th percentiles 0.4-6.7ng/ml; p<0.001). Despite the weight loss, these patients still had a normal BMI (median 23.4kg/m^2^). We did not find any correlation of myostatin prodomain with age, BMI, weight loss or CRP in this cohort. As a second cohort with pro-cachectic activity, we analyzed patients with chronic pulmonary disease (see Table 1 and Table S3). Myostatin prodomain was detectable with the sandwich IRMA in 39 of 44 of these patients (88.6%). The median myostatin prodomain concentration was significantly and markedly higher (median 26.9ng/ml; 25th-75th percentiles 7-100ng/ml; p<0.001, Figure 4) compared to the group of healthy individuals younger than 55 years. The patients in this cohort had a very low BMI (median 17.5kg/m^2^; 25th-75th percentiles 16.9-18.3kg/m^2^; p<0.001), but did not show weight loss over the last 12 months. We did not find any correlation of myostatin prodomain with age, weight loss, BMI or CRP in this cohort.

## Discussion

We established a novel immunoradiometric sandwich assay for the highly specific detection of myostatin prodomain in human serum. The specificity of our assay was proven by several approaches: First, size exclusion chromatography and assessment of myostatin prodomain abundance in 75 subfractions from human serum with high prodomain concentration revealed a single detection peak between 66kDa and 100kDa in size, which was not detected in serum with very low prodomain abundance. This signal peak was most likely produced by the myostatin latent complex of prodomain and ligand (together 80kDa in size), which is the main form of myostatin in serum. Second, myostatin triggered SMAD (CAGA) reporter activation was mainly found in the fractions that also gave a high signal in our IRMA and was not detectable in fractions from the serum with low myostatin abundance. Third, Western-blotting and detection by two different unrelated antibodies revealed that only the signal intense fractions included high amounts of myostatin prodomain as well as myostatin ligand, both of which were not at all detected in fractions outside the signal peak. The serum with the high myostatin prodomain concentration also showed a higher amount of prodomain and ligand in Western-blotting. Lastly, we determined that neither myostatin ligand nor unspecific potentially interfering substances changed the detection of myostatin prodomain in this assay. 

So far, most of the studies that examined myostatin in various disease states used a commercially available competitive immunoassay [20-23,32-38]. By using this assay, it was suggested for example, that serum myostatin levels are increased or decreased in patients with chronic heart failure [20-23], increased or unchanged in diabetes mellitus type 2 and perhaps reduced in adult patients with Pompe disease [32,33,38]. A comparison of these studies revealed that the reported concentrations of serum myostatin were not only highly variable between different cohorts of patients with the same disease, but also between different cohorts of control individuals (without disease) of similar age and sex ratio (mean values between 3.3 ng/ml and 43ng/ml were shown) [22,33]. This competitive immunoassay was designed to detect the precursor molecule promyostatin, which mainly exists in the cell before getting cleaved by furin. Therefore, promyostatin is usually not found in serum [3,4]. Indeed, as revealed by size exclusion chromatography, no signal peak could be detected in 75 subfractions of serum containing high amounts of myostatin latent complex. In addition, the commercial assay did not adequately detect recombinant prodomain or ligand separately or in combination. Therefore, results obtained by the commercial promyostatin assay should be interpreted with great caution, first because of the highly variable results reported in the literature (perhaps reflecting technical shortcomings) and, more importantly, because its specificity is questionable. 

Another assay to quantify myostatin in human serum was a sandwich enzyme bio-assay, which detected the myostatin prodomain [23,39,40]. Compared to our IRMA, different antibodies were used and the specificity of the assay´s antibody combination was not reported. The assay, however, is no longer available since it was discontinued by the company Biovendor that originally invented it. Results obtained demonstrated reduced myostatin prodomain serum levels in 70 heart failure patients versus healthy controls (similar to our results), although the control group was very small (n=10) [23]. Furthermore, myostatin prodomain was found not to be changed in serum in response to resistance exercise in 66 healthy sedentary adults [40]. 71 older patients with stable chronic obstructive pulmonary disease exerted significantly increased myostatin prodomain levels in comparison to 60 healthy control subjects [39]. One group employed Western blotting to detect elevated concentrations of the myostatin prodomain in the serum of 28 patients with dilated cardiomyopathy in comparison to 29 healthy controls [9]. Western blotting, however, is not well suited to analyze the abundance of serum proteins, it cannot be used to analyze a large number of samples and only enables relative but not absolute quantification of serum myostatin prodomain concentrations.

Besides measuring the concentration of the myostatin prodomain, another strategy is to quantify the abundance of the myostatin ligand in serum. Although myostatin prodomain and ligand are produced by cells in an equimolar ratio and the majority of both exists associated with each other as latent complex in serum, it is likely that their degradation is subject to differential regulation and might result in distinct concentrations [1,2]. Earlier studies employing a direct immunoassay were published before it was known that the myostatin ligand circulates bound to other plasma proteins and therefore ignored that its measurement requires acid pre-treatment in order to release it from the latent complex [41-44]. This step, however, often hampers subsequent detection by antibodies and it is unclear if the release of the ligand from the latent complex is complete, i.e. whether really the true amount of myostatin ligand can be quantified by the assay. A specific method to detect the myostatin ligand in human serum has been published recently [24,45]. It was demonstrated, for example, that serum levels of the myostatin ligand are transiently increased in men treated with graded doses of testosterone, but are not increased in sarcopenic men. Other patient cohorts have not been analyzed so far. In addition to the limitations associated with acid pretreatment for detection of the ligand, our prodomain IRMA is likely to be more myostatin specific, since GDF11, which has also been found in serum, exerts a 92% identity to the myostatin ligand and might therefore interfere with its detection [46]. 

We measured the myostatin prodomain concentration in 249 healthy individuals. Interestingly, we observed a slight, but significant decrease of serum prodomain in women compared to men in the age group over 55 years. A decreased myostatin prodomain concentration in women appears logical, since skeletal muscle is the main source of serum myostatin and women have a reduced overall skeletal muscle mass compared to men. A similar trend of increased serum myostatin concentrations in men was observed for the myostatin ligand [24].

We next assessed the prodomain concentration in patients with stable chronic heart failure. Since increased myocardial, skeletal muscle as well as serum concentrations have been detected in animal models of this disease, we expected similar results in humans [5-8]. However, we found a slightly, but significantly *decreased* concentration of myostatin prodomain in the serum of these patients. What could be the reason for this discrepancy, besides the fact that the pathophysiological mechanisms could be different in humans and rodents? First, it should be noted that our heart failure patients showed no signs of cachexia, since the median BMI was indicating overweight and there was no significant weight loss in the cohort. Secondly, the patients we examined in our study had an optimized medical treatment with 96% of the patients receiving beta-blockers and ACE inhibitors or angiotensin II receptor antagonists (ARBs). Since beta-blocker and ACE inhibitors were previously shown to mediate a positive influence on cachexia in heart failure, this treatment might also reduce myostatin expression [47,48]. In agreement with our results, no significant increase in myostatin mRNA was detected in skeletal muscle of heart failure patients with optimized medical therapy compared to healthy controls in a recent heart failure trial [49].

 In order to further test our assay and measure myostatin in patients with cachexia or muscle wasting, we assessed serum prodomain levels in two additional patient cohorts. We found a slightly reduced abundance of myostatin prodomain in the serum of patients with tumor disease of the liver or the gastrointestinal tract and strong recent weight loss. The effects of myostatin in tumor cachexia might depend on myostatin from skeletal muscle, but also on myostatin secreted from tumor cells [12,18,50]. According to our results, the particular cancer patients we examined did not suffer from tumors with myostatin dependent cachexia. The observed downregulation of serum myostatin prodomain in our cancer patients (and the heart failure patients) might in fact constitute a compensatory mechanism to limit muscle loss under these pathological circumstances. In contrast to the cancer patients, patients with pulmonary disease and chronic cachexia/underweight showed a dramatic increase in the serum levels of myostatin prodomain. This is in line with recent data from human cachectic COPD patients with markedly upregulated myostatin mRNA and protein in skeletal muscle and increased myostatin in serum (as detected by Western-blot) and also in line with data demonstrating hypoxia dependent induction of myostatin mRNA in human skeletal muscle cells [11,13].

 Therefore, although other assays have been used to quantify the abundance of myostatin in serum, we provide so far the only assay to detect the myostatin prodomain with proven specificity and without the need for acid- pretreatment of the samples. In addition, we analyze myostatin prodomain abundance in 249 healthy individuals and in 266 patients with different diseases and for the first time demonstrate a profound upregulation of myostatin prodomain in young underweight patients with chronic pulmonary disease (mainly cystic fibrosis). These preliminary results should trigger further analysis of myostatin prodomain serum levels as well as research of the pathophysiological importance of myostatin in this and other patient groups (for example patients with HIV, end stage renal disease or other forms of cancer); in addition, the possibility of therapeutic myostatin blockade should be considered under circumstances of elevated myostatin abundance in the future.

### Limitations of This Study

In this study we introduce an assay that is measuring the myostatin prodomain. As alluded to in the introduction, this is the inactive, N-terminal part of the myostatin precursor molecule, while the C-terminal part is the myostatin ligand, which binds to the activin IIb receptor to inhibit the growth of muscle cells. Although the main form of myostatin ligand in serum occurs in association with its prodomain in the latent complex, the concentration of the ligand could be more closely associated with disease outcome and therefore might be more useful as a biomarker. However, beside the technical difficulties associated with the quantification of the ligand in serum, the prodomain has its own biological role as a strong inhibitor of the myostatin ligand. Therefore, in order to obtain a more complete picture of myostatin signalling in a particular disease state, we suggest that one should assess the serum concentration of both, myostatin ligand and prodomain. Secondly, we used a low BMI (< 20kg/m^2^) as criterion to select chronic pulmonary disease patients with muscle wasting or cachexia. However, BMI is not a good indicator of muscle mass and thus we might have included some patients, although they do not suffer from muscle wasting [51].

Lastly, especially the patients suffering from gastrointestinal or hepatic cancer exerted very low serum concentrations of myostatin prodomain, which were significantly below the values found in healthy individuals. However, these low values are close to the limit of detection and quantification, and therefore our assay might not be sensitive enough to reliably distinct between both groups.

## Supporting Information

Figure S1 **Measurement of** promyostatin in 75 serum fractions with the commercial ELISA. Size-exclusion chromatography of one serum sample with high myostatin prodomain concentration according to our IRMA. The promyostatin concentration in each fraction was determined with the ELISA from *Immundiagnostik*.
(PPTX)

Figure S2 **Quantification of recombinant** **myostatin ligand or prodomain by the commercial ELISA and the IRMA. A** Various concentrations of recombinant myostatin ligand and/or prodomain were determined by the commercially available ELISA from the company *Immundiagnostik*, which was established to measure promyostatin. **B** Various concentrations of recombinant myostatin ligand and/or prodomain were determined by the prodomain specific sandwich IRMA.
(PPTX)

Figure S3 **Myostatin prodomain concentrations as detected by the IRMA in different serum fractions of patients suffering from chronic pulmonary disease.** Size-exclusion chromatography of two serum samples of patients with chronic pulmonary disease and subsequent assessment of myostatin prodomain with the IRMA in serum fractions number 15-60. The maximal detection of myostatin prodomain was found in fraction number 31.
(PPTX)

Materials and Methods S1 (DOCX)

Table S1 **Clinical characteristics of the heart failure patients.**
(DOCX)

Table S2 **Etiology of cancer in this study.**
(DOCX)

Table S3 **Clinical characteristics of the patients with chronic pulmonary disease.**
(DOCX)
