# Supplementary material for: Highly Specific Detection of Myostatin Prodomain by an Immunoradiometric Sandwich Assay in Serum of Healthy Individuals and Patients
Source: PLoS One. 2013 Nov 15;8(11):e80454. doi: 10.1371/journal.pone.0080454 (PMC3829884; doi:10.1371/journal.pone.0080454)
Supplement: Figure S1 — Measurement of promyostatin in 75 serum fractions with the commercial ELISA. Size-exclusion chromatography of one serum sample with high myostatin prodomain concentration according to our IRMA. The promyostatin concentration in each fraction was determined with the ELISA from Immundiagnostik. (PPTX) [file pone.0080454.s001.pptx]

## Slide 1
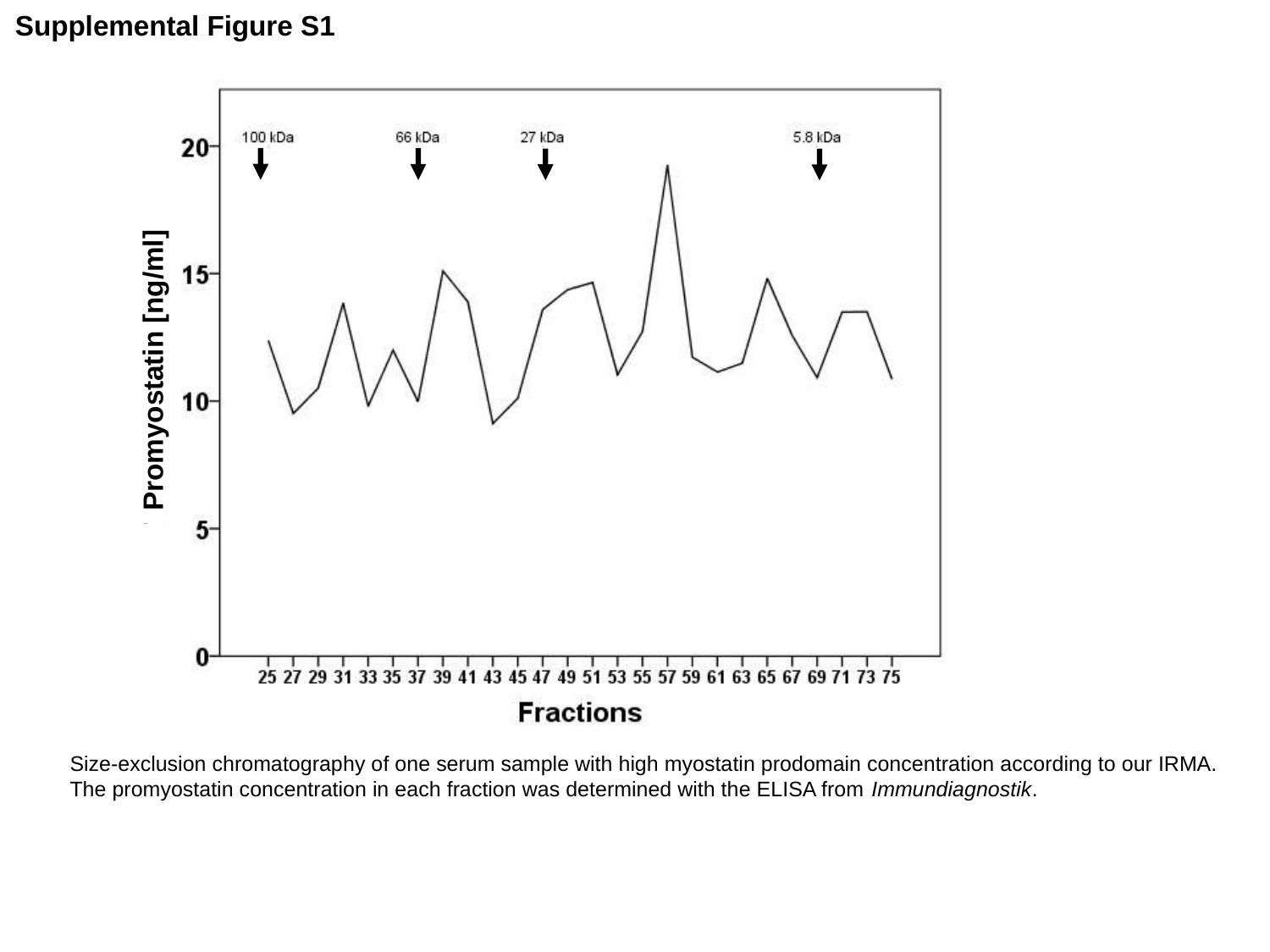

Supplemental Figure S1
Promyostatin [ng/ml]
Size-exclusion chromatography of one serum sample with high myostatin prodomain concentration according to our IRMA.
The promyostatin concentration in each fraction was determined with the ELISA from Immundiagnostik.
