# Supplementary material for: Highly Specific Detection of Myostatin Prodomain by an Immunoradiometric Sandwich Assay in Serum of Healthy Individuals and Patients
Source: PLoS One. 2013 Nov 15;8(11):e80454. doi: 10.1371/journal.pone.0080454 (PMC3829884; doi:10.1371/journal.pone.0080454)
Supplement: Figure S2 — Quantification of recombinant myostatin ligand or prodomain by the commercial ELISA and the IRMA. A Various concentrations of recombinant myostatin ligand and/or prodomain were determined by the commercially available ELISA from the company Immundiagnostik, which was established to measure promyostatin. B Various concentrations of recombinant myostatin ligand and/or prodomain were determined by the prodomain specific sandwich IRMA. (PPTX) [file pone.0080454.s002.pptx]

## Slide 1
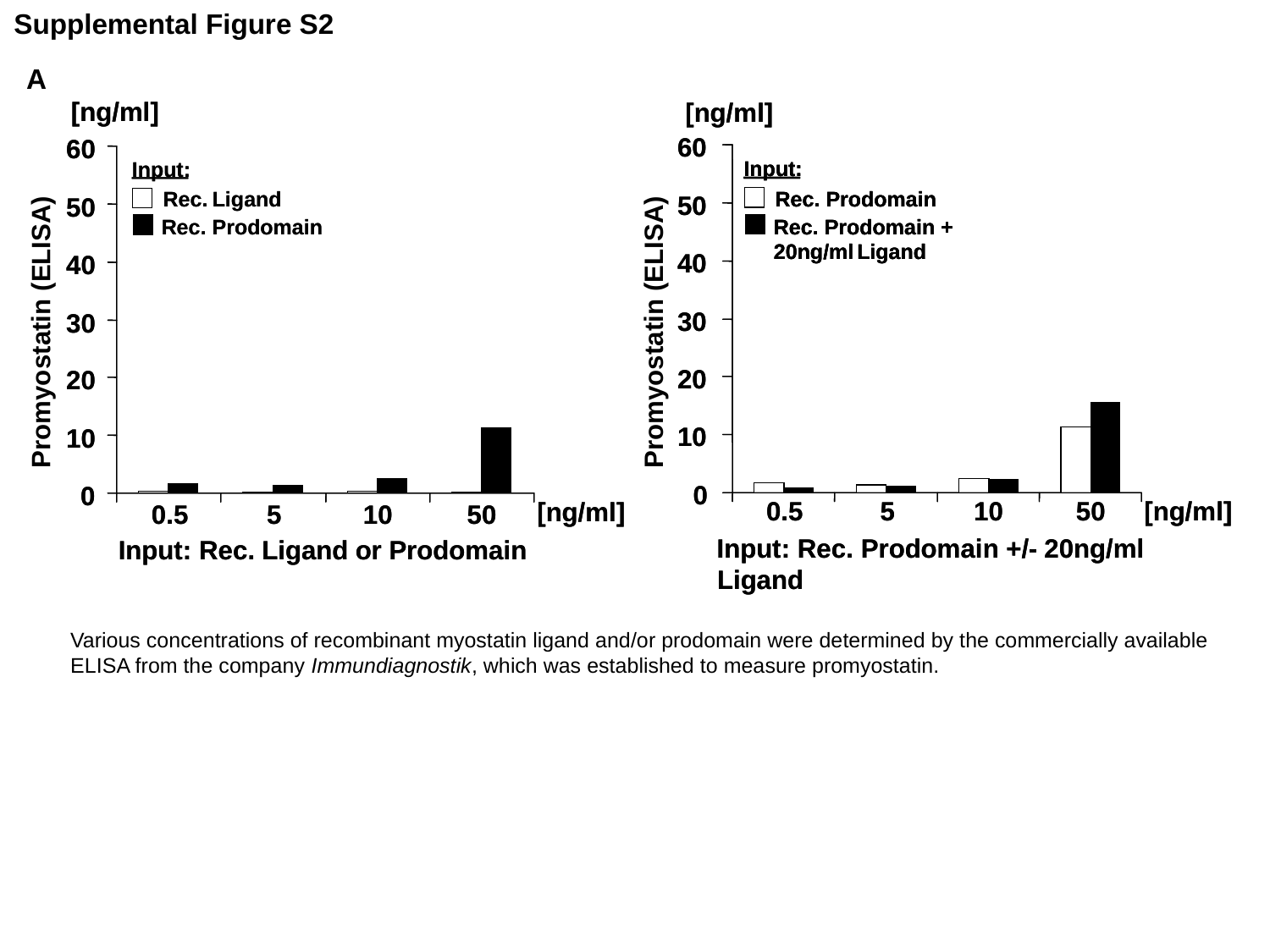

Supplemental Figure S2
A
[ng/ml]
[ng/ml]
[ng/ml]
[ng/ml]
60
60
60
60
Input:
Rec. Prodomain
Rec. Prodomain +
20ng/ml
Ligand
Input:
Input:
Input:
Input:
Rec. Prodomain
Rec. Prodomain
Rec.
Rec.
Ligand
Ligand
50
50
50
50
Rec. Prodomain +
Rec. Prodomain +
Rec. Prodomain
Rec. Prodomain
20ng/ml
20ng/ml
Ligand
Ligand
40
40
40
40
30
30
30
30
Promyostatin (ELISA)
Promyostatin (ELISA)
20
20
20
20
10
10
10
10
0
0
0
0
0.5
0.5
5
5
10
10
50
50
[ng/ml]
[ng/ml]
[ng/ml]
[ng/ml]
0.5
0.5
5
5
10
10
50
50
Input: Rec. Prodomain +/
Input: Rec. Prodomain +/
-
-
20ng/ml
20ng/ml
Input: Rec.
Input: Rec.
Ligand
Ligand
or
or
Prodomain
Prodomain
Ligand
Ligand
Various concentrations of recombinant myostatin ligand and/or prodomain were determined by the commercially available
ELISA from the company Immundiagnostik, which was established to measure promyostatin.

## Slide 2
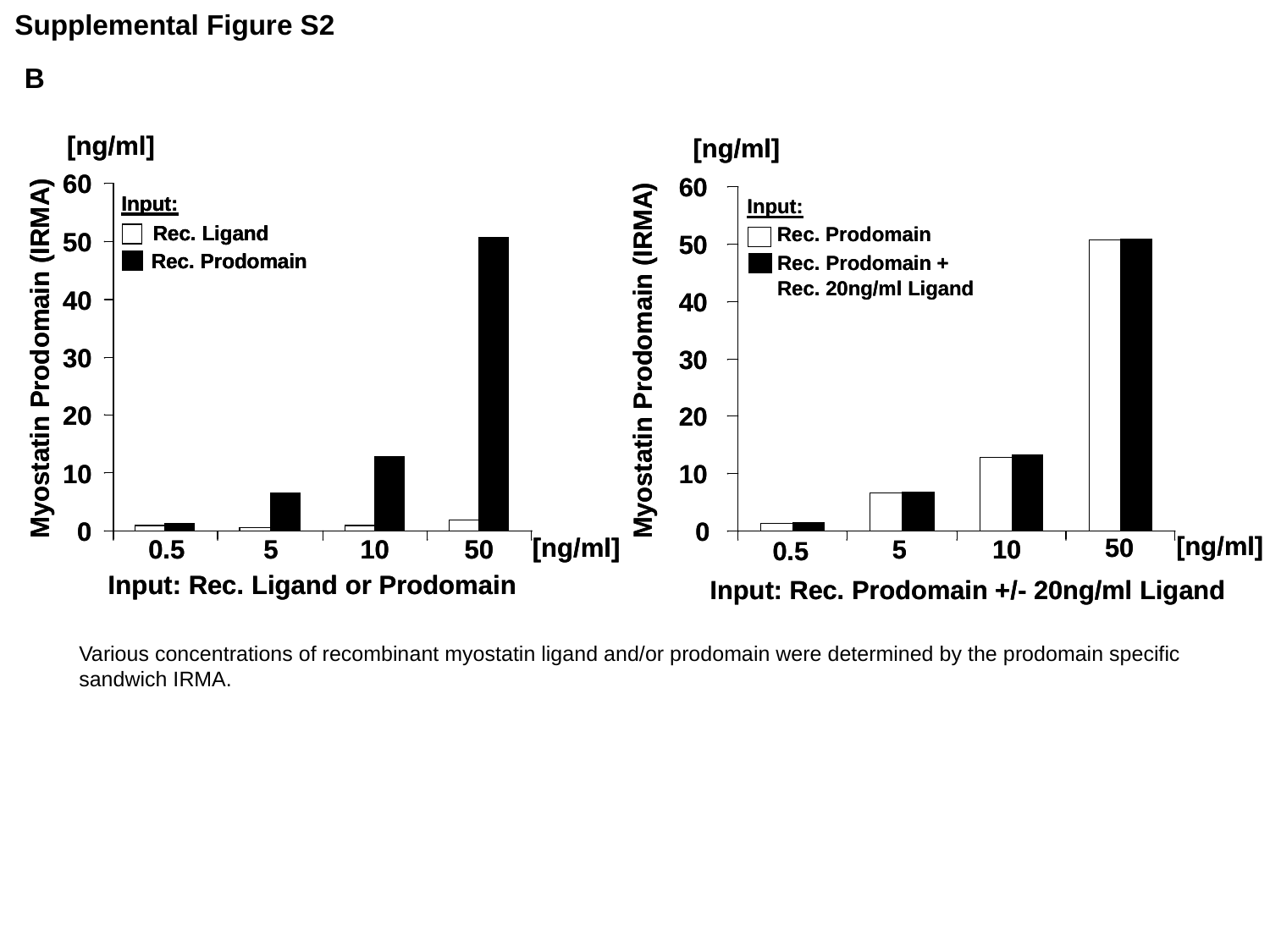

Supplemental Figure S2
B
Various concentrations of recombinant myostatin ligand and/or prodomain were determined by the prodomain specific
sandwich IRMA.
