# Supplementary material for: Highly Specific Detection of Myostatin Prodomain by an Immunoradiometric Sandwich Assay in Serum of Healthy Individuals and Patients
Source: PLoS One. 2013 Nov 15;8(11):e80454. doi: 10.1371/journal.pone.0080454 (PMC3829884; doi:10.1371/journal.pone.0080454)
Supplement: Figure S3 — Myostatin prodomain concentrations as detected by the IRMA in different serum fractions of patients suffering from chronic pulmonary disease. Size-exclusion chromatography of two serum samples of patients with chronic pulmonary disease and subsequent assessment of myostatin prodomain with the IRMA in serum fractions number 15-60. The maximal detection of myostatin prodomain was found in fraction number 31. (PPTX) [file pone.0080454.s003.pptx]

## Slide 1
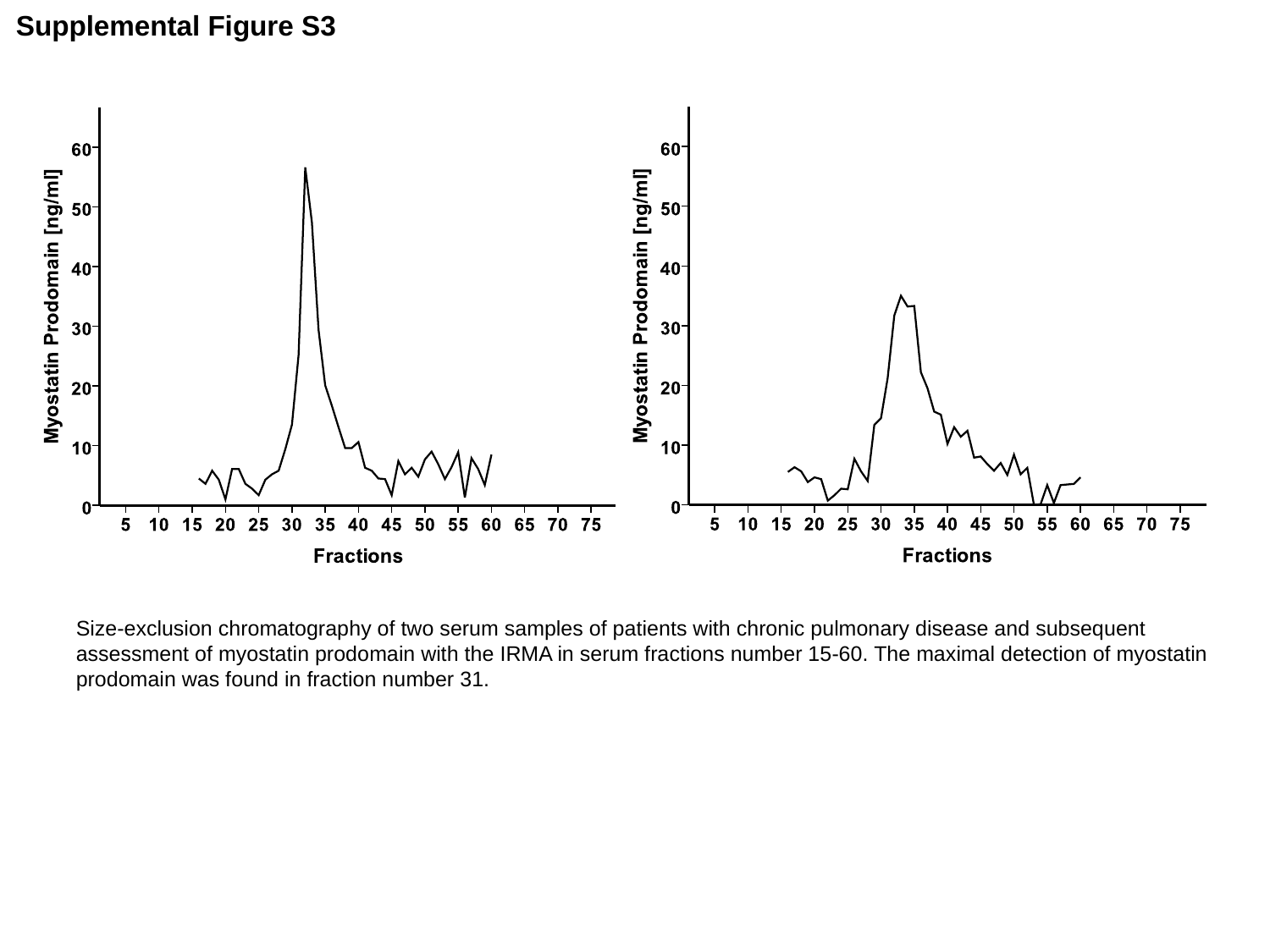

Supplemental Figure S3
Size-exclusion chromatography of two serum samples of patients with chronic pulmonary disease and subsequent
assessment of myostatin prodomain with the IRMA in serum fractions number 15-60. The maximal detection of myostatin
prodomain was found in fraction number 31.
