# Supplementary material for: Highly Specific Detection of Myostatin Prodomain by an Immunoradiometric Sandwich Assay in Serum of Healthy Individuals and Patients
Source: PLoS One. 2013 Nov 15;8(11):e80454. doi: 10.1371/journal.pone.0080454 (PMC3829884; doi:10.1371/journal.pone.0080454)
Supplement: Materials and Methods S1 — (DOCX) [file pone.0080454.s004.docx]

**Supplemental Material and Methods**

*Additional Materials*

Recombinant human myostatin propeptide (cyt-448-b) was purchased from Prospec.

*Size-exclusion chromatography*

In order to assess the specificity of the assay, we fractionated two serum samples of heart failure patients (one with high, 1629ng/ml, and one with low myostatin concentration, 1.37ng/ml, as determined with our IRMA) by size exclusion chromatography on a sephadex G-100 column. TRIS buffer (20mmol/L TRIS, 150mmol/L sodium chloride, 1g/L sodium azide, 5g/L bovine serum albumin, pH 7.0) was used to equilibrate the column. 1ml serum was loaded onto the column and collected in 75 fractions. Subsequently, myostatin prodomain was measured in each fraction by the sandwich IRMA. Blue dextran (2000kDa; Pharmacia) was used to determine the columns void volume, bovine serum albumin (66kDa), recombinant myostatin propeptide (27.8kDa) and insulin (5.8kDa; Lilly) were used as molecular mass indicators.

*P (CAGA)_12_-luciferase reporter assay*

HEK293 cells (ATCC) were transfected with the pGL3(CAGA)_12_ reporter plasmid and plated on 48-well plates. 25µl of the various fractions (no-acid treatment) and 50µl of the acid/neutralization treated fractions were added per well (supplemented to a total volume of 300µl with culture-medium) and cultured subsequently for 24 hours. The HEK293 cells were then lysed and luciferase activity was assayed. The results were normalized for protein content per well, which was determined by the Micro BCA^TM^ Protein Assay Kit (Thermo Scientific) and were calculated for each sample as the ratio of the specific luciferase activity (per µg protein) with and without acid activation. Acid activation was conducted by adding 50µl of 1N HCl to 100µl of the respective serum fraction and incubation for 10 minutes. Next, 50µl of 1.2N NaOH, 0.5M Hepes solution was added for neutralization.

*Western-blot*

To further assess the specificity of the assay we analyzed selected fractions of both serum samples by Western-blot. We used a monoclonal anti-myostatin propeptide antibody (MAB7881, R&D Systems) for detection of the myostatin prodomain and a polyclonal anti-myostatin antibody (AB3239, Millipore) for detection of the myostatin ligand.

*Serum samples*

To evaluate the preanalytic performance of the myostatin prodomain IRMA we used serum samples from apparently healthy persons (n=13, 4 males; ages 18-55 years) and pooled serum samples from apparently healthy blood donors (n=7, ages 20-65 years).

*Healthy individuals*

We collected serum from apparently healthy blood donors from the blood donor center at Hannover Medical School. We included 63 blood donors aged 18-55 years. We also collected serum from 186 blood donors aged at least 56 years. More information regarding the healthy individuals is displayed in Table 1.

*Patients with chronic heart failure*

Myostatin prodomain was determined in 169 serum samples of patients with stable chronic heart failure. The patients were recruited from the outpatient arrhythmia clinic at Hannover Medical School. Patients with decompensated heart failure, critical illness during the last month, active cancer, severe kidney disease or chronic inflammatory disease were excluded from the study. We determined the BMI and the serum levels of creatinine, CRP, BNP and GDF15 (please see Table 1 and Supplemental Table S1 for further information). Patients were re-evaluated (BMI assessment) during a follow-up visit 6 months later. 20 patients (11.8%) were lost to follow up.

*Patients with gastrointestinal tumors*

We quantified the serum concentration of myostatin prodomain in 53 patients with cancer of the gastrointestinal tract or the liver, who reported unwanted weight loss of at least 4% in the last 12 months. The patients were recruited from the outpatient gastro-oncological clinic at Hannover Medical School. Further information can be found in Table 1 and Supplemental Table S2.

*Patients with chronic pulmonary disease*

We determined the serum concentration of myostatin prodomain in 44 patients with chronic pulmonary disease, who presented with a BMI below 20kg/m^2^. The patients were recruited from the outpatient pneumological clinic at Hannover Medical School. Patient characteristics are listed in Table 1 and Supplemental Table S3.
