# Supplementary material for: Highly Specific Detection of Myostatin Prodomain by an Immunoradiometric Sandwich Assay in Serum of Healthy Individuals and Patients
Source: PLoS One. 2013 Nov 15;8(11):e80454. doi: 10.1371/journal.pone.0080454 (PMC3829884; doi:10.1371/journal.pone.0080454)
Supplement: Table S1 — Clinical characteristics of the heart failure patients. (DOCX) [file pone.0080454.s005.docx]

|  | **Heart Failure**  **N=169** |
| --- | --- |
| **Etiology** |  |
| ICM | 58.3 |
| DCM | 30.4 |
| other | 11.3 |
| **Classification** |  |
| NYHA II | 69.2 |
| NYHA III | 30.8 |
| **Medication** |  |
| ASS | 53.3 |
| Oral anticoagulant | 34.7 |
| ACE-inhibitor or ARB | 95.3 |
| Beta-Blocker | 95.3 |
| Furosemide | 53.3 |
| Spironolactone | 50.9 |
| Statins | 68.9 |

**Supplemental Table S1:**

**Clinical characteristics of the heart failure patients.**

Data are displayed in % of all heart failure patients. ICM denotes ischemic and DCM denotes dilative cardiomyopathy. NYHA II and III indicate New York Heart Association Class II and III. ARB denotes angiotensin II receptor antagonist.
