# Supplementary material for: Highly Specific Detection of Myostatin Prodomain by an Immunoradiometric Sandwich Assay in Serum of Healthy Individuals and Patients
Source: PLoS One. 2013 Nov 15;8(11):e80454. doi: 10.1371/journal.pone.0080454 (PMC3829884; doi:10.1371/journal.pone.0080454)
Supplement: Table S2 — Etiology of cancer in this study. (DOCX) [file pone.0080454.s006.docx]

**Supplemental Table S2:**

**Etiology of cancer in this study.**

Displayed in % of all gastrointestinal or hepatic cancer patients.

|  | **Gastrointestinal/Hepatic**  **Cancer**  **N=53** |
| --- | --- |
| **Type of Cancer** |  |
| Hepatocellular | 28.3 |
| Pancreatic | 18.9 |
| Colo-rectal | 18.9 |
| Gastric | 15.1 |
| Oesophageal | 9.4 |
| Other | 9.4 |
