# Supplementary material for: Highly Specific Detection of Myostatin Prodomain by an Immunoradiometric Sandwich Assay in Serum of Healthy Individuals and Patients
Source: PLoS One. 2013 Nov 15;8(11):e80454. doi: 10.1371/journal.pone.0080454 (PMC3829884; doi:10.1371/journal.pone.0080454)
Supplement: Table S3 — Clinical characteristics of the patients with chronic pulmonary disease. (DOCX) [file pone.0080454.s007.docx]

**Supplemental Table S3:**

**Clinical characteristics of the patients with chronic pulmonary disease.**

Data are presented as the percentage of all patients with chronic pulmonary disease or the median (25^th^-75^th^ percentile). FEV1% stands for forced expiratory volume in 1 second in percent of the value expected in a healthy person.

|  | **Chronic Pulmonary Disease**  **N=44** |
| --- | --- |
| **Disease** |  |
| Cystic Fibrosis | 57.1% |
| Emphysema | 14.3% |
| Pulmonary Fibrosis | 11.9% |
| COPD | 9.5% |
| Bronchiectasis | 4.8% |
| Sarcoidosis | 2.4% |
| **Severity of Disease** |  |
| FEV1% | 22.5 (19-28) |
